# Intratendinous Air Phenomenon: A New Ultrasound Marker of Tendon Damage?

**DOI:** 10.3389/fphys.2017.00570

**Published:** 2017-08-03

**Authors:** Saulius Rutkauskas, Vidas Paleckis, Albertas Skurvydas, Danguole Satkunskiene, Marius Brazaitis, Audrius Snieckus, Neringa Baranauskiene, Ruslanas Rancevas, Sigitas Kamandulis

**Affiliations:** ^1^Institute of Sports Science and Innovation, Lithuanian Sports University Kaunas, Lithuania; ^2^Department of Radiology, Lithuanian University of Health Sciences Kaunas, Lithuania

**Keywords:** vacuum phenomenon, exercise-induced muscle damage, metabolic fatigue, drop jumps, tendons

## Abstract

**Purpose:** To explore the presence of intratendinous air in physically active males after different types of strenuous physical exercise.

**Materials and Methods:** To detect foci (air bubbles) in the quadriceps femoris tendon (QFT) and the proximal and distal parts of the patellar tendon, ultrasound examination was performed under two conditions: (1) after high-intensity cycling on a cycle ergometer (metabolic); (2) after 200 drop jumps (exercise-induced muscle damage). Based on the results of these two interventions, the presence of air in the tendons after 100 drop jumps was examined further with frequently repeated ultrasound measurements.

**Results:** Foci were detected in exercise-induced muscle damage. Twenty-three of Sixty investigated tendons (38.3%) were observed to contain hyperechoic foci after 100 drop jumps. QFT foci were present in 13/23 cases (56.5%). The location of foci in the QFT was mostly lateral and centro-lateral (76.9%). The foci disappeared completely between 40 and 180 min after completing 100 drop jumps.

**Conclusions:** The presence of intratendinous air seems related to high-magnitude, high-force, high-strain exercise of the particular tendon areas. It might represent the stress response of tendons to overload condition.

## Introduction

The vacuum phenomenon is currently defined as the accumulation of air in natural cavities such as synovial joints and intervertebral discs (Jordanov and Block, 2010; D'Anastasi et al., 2011; Motamedi et al., 2014). It has been demonstrated that air can be found in hip (Fairbairn et al., 1995; Liu et al., 2015; Schröder et al., 2016), shoulder (Ito et al., 2008; Jordanov and Block, 2010), sternoclavicular (Ito et al., 2008), metacarpophalangeal (Malghem et al., 2011), temporomandibular (Moncada et al., 2008), and knee joints (Jordanov and Block, 2010) and in degenerative intervertebral discs (Stallenberg et al., 2001; D'Anastasi et al., 2011; Feng et al., 2011; Wadhwa et al., 2016). The features of the vacuum phenomenon detected using different imaging techniques have been discussed widely (Coulier, 2004; Gohil et al., 2014; Yanagawa et al., 2016). Fick was the first to identify the vacuum phenomenon on radiographic images (Fick, 1919). Since then it has been demonstrated that computed tomography (CT) has a higher sensitivity than radiography and magnetic resonance imaging (MRI) for evaluating the presence of intra-articular air in large joints and vertebral discs (Ito et al., 2008; Moncada et al., 2008; Jordanov and Block, 2010; D'Anastasi et al., 2011; Motamedi et al., 2014; Wadhwa et al., 2016), while radiography and ultrasonography are more sensitive than CT and MRI for evaluating the presence of air in small joints (Malghem et al., 2011).

On ultrasound, intra-articular air is seen as free-floating bright foci of variable size that persist for no more than 30 min (Malghem et al., 2011). During routine performance of musculoskeletal ultrasound, similar foci were surprisingly discovered in the tendons. These foci were usually present at the insertional sites of the tendon, and had a cloud-like or elongated form. The patients showing this phenomenon were of different ages and had different ultrasound pathologies (e.g. meniscus tear or pre-patellar bursitis) or were without abnormal findings. Notably, these bright spots in the tendon were found in almost all basketballers after a short period of training (unpublished data).

Having considered that the tendons are affected by an increased physical load, we suggested a hypothetical “intratendinous air phenomenon.” To explore the conditions under which intratendinous air was detected, we applied several strenuous interventions aimed at inducing different types of neuromuscular adaptation in physically active non-athletes. In addition, we evaluated the localization and dynamic changes of the foci. To our knowledge, this is the first scientific study investigating the phenomenon of air in the tendons.

## Methods

### Subjects

The subjects were randomly recruited healthy males involved in different recreational activities (such as jogging or cycling) of light to moderate intensity at a frequency of 1–2 times per week. They were requested not to exercise for at least 1 week before the study and do not take any medication or dietary supplement during the entire experimental period. None of the subjects had any history of any musculoskeletal disorders or other disease (such as metabolic, inflammatory) that could potentially affect the lower musculature or leg tendons. Ultrasound examination of the knee joints was performed before study and subjects with any signs of pathology (e.g., effusion or tendinopathy) were excluded. All the subjects were free of medications that could directly or indirectly affect tendons, neither before not during the study. Subjects involved in different interventions did not overlap. The mean age and body mass of subjects in interventions I, II, and III were 27.1 ± 4.8 years and 79.9 ± 5.2 kg (*n* = 6), 29.8 ± 9.3 years and 81.7 ± 5.8 kg (*n* = 10), and 25.4 ± 5.2 years and 78.7 ± 4.6 kg (*n* = 10), respectively. Before the study commenced, all subjects read and signed a written informed consent that was consistent with the principles outlined in the Declaration of Helsinki. Approval for the study of human participants was received from the Regional Ethics Committee before data collection.

### Ultrasound examination

**Figure 1:**
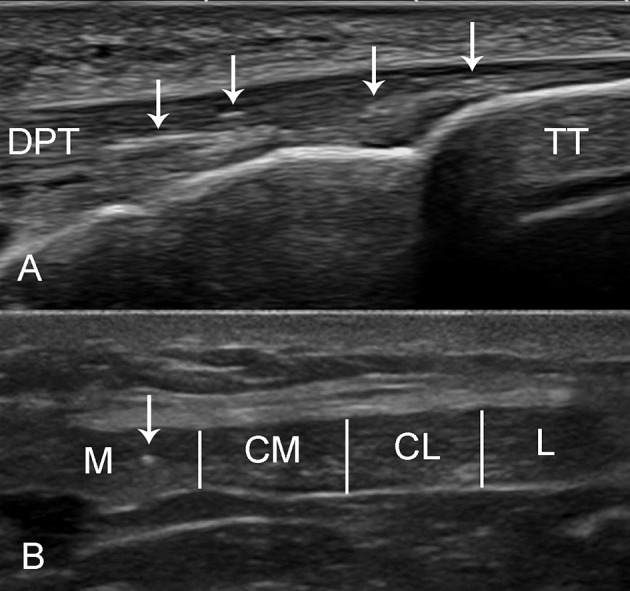
Ultrasound imaging of the distal patellar tendon (DPT). **(A)** The long axis of the DPT of a 34-year-old male was evaluated with Toshiba linear ultrasound probe (5–14 MHz). The bright foci (arrow) can be seen at the tendon insertion into the tibial tuberosity (TT). **(B)** The short axis of the patellar tendon (Mindray) was divided into four zones depending on the localization of the bright spots: medial (M), centro-medial (CM), centro-lateral (CL), and lateral (L). A small bright focus can be seen in the medial region. The patellar tendon appears hypoechoic because of anisotropy.

The ultrasound examination was performed using a Mindray M7 Diagnostic ultrasound system (Shenzhen Mindray Bio-Medical Electronics Co. Ltd, China) with a linear L14-6Ns transducer (10-12 MHz) by a radiologist with 10 years' experience of ultrasound assessment of the musculoskeletal system. To determine that finding did not depend on ultrasound device used, several subjects were assessed with more sophisticated Toshiba ultrasound equipment (linear transducer PLT1005BT, range 5–14 MHz, basic frequency 10 MHz, MSK preset). No significant effect of the device was found on the foci distinguishability. The presence of intratendinous foci was evaluated longitudinally and transversally (Figure 1A) with the usual musculoskeletal imaging preset for the quadriceps femoris tendon (QFT), and the proximal (PPT) and distal (DPT) parts of the patellar tendon. The participant was in a supine position with their knees supported and flexed. The examination was performed at the most appropriate degree of knee flexion (approximately 30–40°) at which the tendon is taut with well-defined margins and is homogeneous. For each subject, we acquired at least one image for each tendon at separate locations. Total of 36 tendons ultrasound assessment in Intervention I (six subjects, two sides per subject, three locations per side, two times), 40 tendons in Intervention II (ten subjects, two sides per subject, two locations per side, four times) and 60 tendons in Intervention III (ten subjects, two sides per subject, three locations per side, 16 times) were performed for each person. This resulted in total of 1,192 images for entire study. The images were stored and later analyzed independently by two radiologists. The presence of bubbles was confirmed if both radiologists approved at least one separate sub-millimeter-sized focus spot. The localization of the bright spots in each tendon was divided into four zones: medial, centro-medial, centro-lateral, and lateral (Figure 1B).

### Study design

To test for the presence of foci in tendons, an ultrasound examination was performed under two conditions: (1) after maximum-intensity cycling on a cycle ergometer (extreme metabolic fatigue, intervention I); and (2) after 200 drop jumps (extreme muscle damage, intervention II). Based on the results of these two interventions, the presence of air in the tendons was further examined after 100 drop jumps using frequently repeated ultrasound measurements (intervention III). In addition to the ultrasound measurements, typical markers of exercise-induced muscle damage [muscle strength decline, increased creatine kinase (CK) activity] and metabolic fatigue (performance decline, increased lactate) were assessed. The equipment and techniques used to measure the selected variables were the same as those used in previous studies (Kamandulis et al., 2012).

**Intervention I** consisted of 6 × 30 s bouts of maximum-intensity cycling with 4 min rest after each bout; the average power every 5 s was used for the analyses. The resistance was set at 7.5% of body weight on a Monark 828E cycle ergometer (Monark Exercise AB, Sweden). The exercise was preceded by a 10-min warm-up on a cycle ergometer at a workload of 100 W and pace of 60 rpm. The presence of hyperechoic foci was evaluated after the warm-up and after each cycling bout. Assessment of blood lactate was performed before and immediately after the maximum-intensity cycling.

**Intervention II** consisted of 200 drop jumps, where the subjects jumped down from a height of 0.5 m, landing on the force platform (Powertimer Testing System, Newtest, Finland) with a countermovement up to 90° knee angle immediately followed by a maximal jump upward and again landing on the platform. There were 30-s intervals between jumps and 20-min intervals after every 50 jumps to minimize changes in the energy metabolites that can affect muscle function. Maximal voluntary contraction (MVC) peak torque of the knee extensor muscles was measured when the subjects were sitting upright in a dynamometer chair (System 3; Biodex Medical Systems, Shiley, New York) with the knee joint positioned at an angle of 110° (180° represents full knee extension) for the isometric torque measurements. The peak MVC was maintained for ~3 s before relaxation and was measured twice; the larger value was used in the analysis. Two additional measurements of MVC were performed with the maximal electrical impulse (250 ms, 100 Hz; MG 440; Medicor, Budapest, Hungary) superimposed on the voluntary contraction to assess the central activation ratio. Electrical stimuli to the quadriceps muscle were delivered through surface electrode (9 × 18 cm) in trains of monopolar square wave pulses of 1 ms duration (voltage, 120–150 V). The ultrasound examination of the quadriceps tendon and the distal part of the patellar tendon of both legs and the muscle function assessment of the right knee extensor muscles were performed before the intervention, immediately after 100 or 200 jumps and 24 h after the jumps. Evaluation of CK activity in the blood was performed before and 24 h after the intervention.

**Intervention III** consisted of 100 drop jumps performed using the same method and equipment as for intervention II, excluding the MVC and electrical stimulation. There were 30-s intervals between drop jumps and 2-min rest intervals after 2, 4, 6, 8, 10, 15, 20, 25, 50, 75, and 100 drop jumps for the ultrasound foci examination. The ultrasound measurements were repeated every 20 min after completion of 100 drop jumps until the disappearance of the foci.

### Statistical analysis

Data are presented as the mean ± SD. A one-way analysis of variance (ANOVA) for repeated measures was used to determine the effects of the intervention on dependent variables. When the dependent variable was not normally distributed, the Mann–Whitney *U* or Wilcoxon *W* test was used to compare groups. The level of significance was set at 0.05. All calculations were performed using IBM SPSS v. 20 (IBM, Armonk, NJ).

## Results

### Intervention I

Compared with the baseline, the peak power had dropped by 69.7 ± 8.1% at the end of the last cycling bout (*p* < 0.05). The blood lactate concentration increased from 1.5 ± 0.3 mmol/L to 19.2 ± 1.4 mmol/L 3 min after completion of the last cycling bout.

Hyperechoic foci were identified in seven of 36 tendons (19.4%) and usually were observed as separate spots. They were noticed in three of seven DPTs (42.8%). No foci were observed in any tendon in one of the subjects.

### Intervention II

Declines in MVC of 25.8 ± 10.2% and 30.6 ± 11.6% were observed after 100 and 200 drop jumps, respectively (*p* < 0.05 for both time points). These declines remained significant (25.1 ± 8.7%, *p* < 0.05) 24 h after the jumps. Central activation ratio decreased from 97.2 ± 2.8% at baseline to 92.4 ± 7.5% after 100 drop jumps (*p* > 0.05) and 88.4 ± 10.1% after 200 drop jumps (*p* < 0.05, compared to baseline), while remained 90.3 ± 9.2% at 24 h after 200 drop jumps (*p* < 0.05, compared to baseline). The blood CK activity was 153.6 ± 100.2 IU·L^−1^ before the jumps and significantly higher (1718.4 ± 954 IU·L^−1^; *p* < 0.05) 24 h after the jumps. After 200 jumps, slight changes in the ultrasound were identified in some subjects: one had small effusions on both sides of the deep infrapatellar bursas, and in the another subject increased effusions were noticed in both suprapatellar bursas. After 24 h, all these changes had disappeared.

Intratendinous foci were observed in 21 of 40 tendons (52.5%, Table 1). Table 2 shows that there was a significant asymmetry of the left and right knees' QFT and DPT: bright spots were observed significantly more frequently in the right knee than the left knee tendons. No foci were observed in any tendon in one of the subjects.

**Table 1:** The presence of foci in the quadriceps femoris tendon (QFT), proximal patellar tendon (PPT), and distal patellar tendon (DPT).

**Intervention**	**Number of participants**	**Number of tendons**	**Appearance of intratendinous air**					
			**Right QFT**	**Right PPT**	**Right DPT**	**Left QFT**	**Left PPT**	**Left DPT**
I	6	36	1	1	1	1	1	2
II	10	40	8	–	7	4	–	2
III	10	60	7	1	4	6	1	4
Total	26	136	16	2	12	11	3	8

**Table 2:** The localization of intratendinous air collection according to the different insertional zones of quadriceps femoris tendon (QFT), proximal insertion of patellar tendon (PPT) and distal insertion of patellar tendon (DPT).

**Tendons**	**Experiments**	**Right side**				**Left side**			
		**Lateral**	**Centro-later**	**Centro-medial**	**Medial**	**Medial**	**Centro-medial**	**Centro-lateral**	**Lateral**
QFT	1	–	–	1	–	–	–	–	–
	2	2	7	5	–	–	–	3	1
	3	4	6	2	–	–	1	6	4
	Total	6	13	8	–	–	1	9	5
PPT	1	–	–	2	–	–	–	–	
	3	–	1	1	1	1	1	–	–
	Total	–	1	3	1	1	1	–	–
DPT	1	–	–	1	1	–	–	2	1
	2	2	6	7	7	2	2	–	–
	3	1	3	3	2	1	3	3	2
	Total	3	9	11	10	3	5	5	3

### Intervention III

Twenty-three of 60 investigated tendons (38.3%, Table 1) contained hyperechoic foci. They were present in 13/23 QFT (56.5%), with the second most frequent localization of foci being the DPT (34.8%). No foci were detected in two subjects.

Our results indicated that foci in the QFT were mostly located in the lateral and centro-lateral areas (76.9%) (Table 2); 80% of the PPT bright spots were in a medial or centro-medial location, and 66.7% of those in the DPT were in centro-medial and centro-lateral locations.

**Figure 2:**
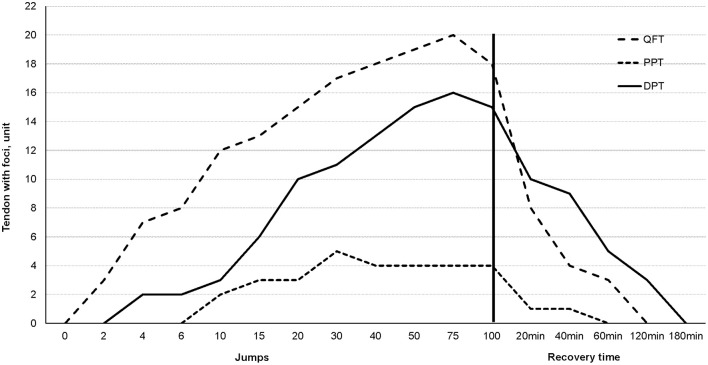
A diagram representing the expression of intratendinous foci during the third intervention. The vertical black line indicates the end of the exercise and the beginning of relaxation.

**Figure 3:**
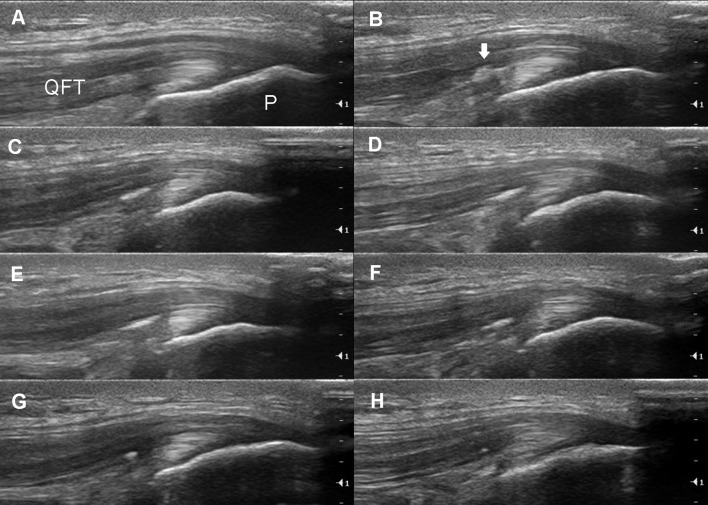
Longitudinal ultrasound imaging of the same subject. **(A)** The normal right quadriceps femoris tendon (QFT) and its insertion into the patella (P). **(B)** Intratendinous collections of bright foci (arrow) can already be clearly visualized after two jumps. Dynamic changes in the intratendinous foci can be seen after 15 **(C)**, 40 **(D)**, 75 **(E)**, and 100 **(F)** jumps and 20 min **(G)** and 40 min **(H)** after exercise.

Figures 2, 3 illustrate the dynamics of intratendinous foci development. The graph shows that foci occur more quickly and disappear more rapidly in the QFT than in the DPT. It also indicates that after completion of 100 jumps, a significant decline in foci occurs until the foci disappear completely at between 40 and 180 min.

## Discussion

Overload of tendons is one of the main causes of degenerative tendinopathy, especially at the insertional sites (Selvanetti et al., 1997; Maffulli et al., 2003; Sharma and Maffulli, 2005; Killian et al., 2012). Insertional tendinopathy is described as a separate pathology that plays a crucial role in the diagnosis and treatment of patients (Fenwick et al., 2002; Killian et al., 2012; Nissman and Dahiya, 2014; Rees et al., 2014). It has been reported that the majority of tendon ruptures are the result of a long-lasting tendinopathy (Maffulli et al., 2003; Xu and Murrell, 2008; Rees et al., 2014; Cook et al., 2016). Our results showed that almost all intratendinous foci were observed at the tendon attachment sites. This suggests that the presence of intratendinous foci could be the initial stage of tendon damage.

Intratendinous foci formation can be explained by comparison with the intra-articular vacuum phenomenon (Coulier, 2004; Gohil et al., 2014; Yanagawa et al., 2016). Tendons (like intra-articular spaces) can be described as a separate anatomical entity with a single pressure. Therefore, excessive overload could cause a decrease in intratendinous pressure and the appearance of air bubbles. It is difficult to explain why the enthesis of the tendon can bear tensile forces four times greater than those withstood by the mid-portion (Wang, 2006), but it seems that a larger workload is needed for intratendinous air formation. On the well-known stress–strain curve, the slope of the linear region is referred to as the Young's modulus of the tendon (Wang, 2006). Microscopic damage to the tendon appears when tendons are stretched by more than 4%, while macroscopic failure occurs with stretching over 8–10%. It is difficult to determine the level of stretching that would result in the occurrence of intratendinous air. Nevertheless, it seems that stretching during eccentric exercise could be related to tendinopathy, and intratendinous air appears to be one of the first ultrasound signs detecting the sensitivity of tendons to overload.

**Figure 4:**
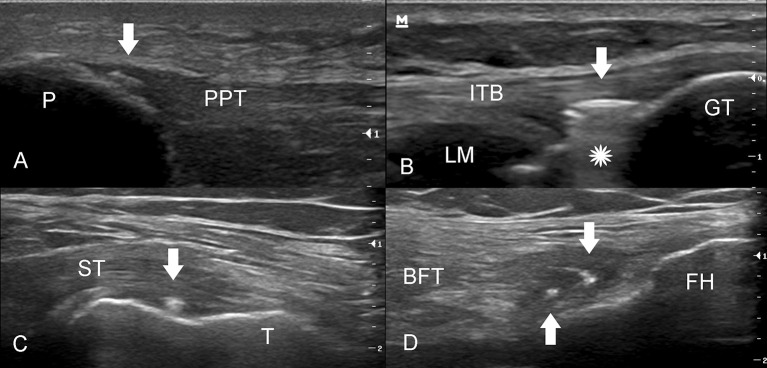
Various attachments of the knee tendons showing signs of intratendinous air (white arrows). **(A)** The insertion of the proximal patellar tendon (PPT) into the patella (P). The small hyperechogenic focus (arrow) appears with blurred contours and without any acoustic shadow. **(B)** The long axis of the insertion of the iliotibial band (ITB). The arrow shows the collection of the intratendinous air with a down-ring artifact (star) (Video 4b). GT, Gerdy's tubercle; LM, lateral meniscus. **(C)** The long axis of semimembranosus tendon (ST) insertion into the posterior aspect of the medial condyle of tibia (T). **(D)** The common insertion of the biceps femoris tendon (BFT) and lateral collateral ligament (not shown) in the fibular head (FH).

Our observations support other evidence that this phenomenon is really not an artifact, but the presence of intratendinous air. Firstly, the foci have the characteristic features of air (blurred contours, no acoustic shadow and a down-ring artifact in ultrasound) (Figures 4A,B). Secondly, the size, form and location of intratendinous air appear to be very dynamic and dependent on the workload at the tendon insertions. Thirdly, on repeated ultrasound scan the hyperechoic foci totally disappear. And fourthly—the same features of air accumulation we have observed in several participants with different ultrasound machines—Mindray and Toshiba (Figure 1A).

We observed formation of separate sub-millimeter-sized air foci in some subjects even after only two drop jumps in QFT (Figures 2, 3.) Further jumping caused the increase in number of foci in a localized cloud-like collection. It was still possible to discern separate air bubbles, but the majority of the bubbles have already been merged into a single larger focus. The collection of air bubbles stabilizes between thirty-seventy five jumps, depending on the tendon. Interesting to note, that in some subjects the commencement of a decrease in air foci in QFT and PPT occur while an intensive workload is still present. The dissipation of air bubbles continued after the exercise quite rapidly. As observed from our experiments, the time for air disappearance from the tendons can be as fast as 20 to 180 min, and no air bubbles were visible 24 h after exercise.

Intratendinous air can be multi-local and was observed not only in the DPT (Figure 1), QFT (Figure 3) and PPT (Figure 4A) but also in other tendons (unpublished data), e.g., the iliotibial band (Figure 4B), semimembranosus tendon (Figure 4C) and the biceps femoris tendon (Figure 4D). It also appears that intratendinous air may be found in other major tendons including the Achilles and plantar fascia tendons, and it is also possible that there is intraligamentous air. Differential diagnosis of air bubbles could be the insertional calcific tendinopathy and enthesophytes. The latter changes usually present with acoustic shadow and are stable during long time observation.

In the present study, we observed the typical signs of exercise-induced muscle damage after drop jumps: a prolonged decrease in MVC (within 24 h) and a large increase in plasma CK activity (>10 times). It is well established that eccentric (lengthening) contraction loads can partially damage the structures of sarcomeres, myofibrils or other cytoskeletal proteins and the sarcolemma and disrupt Ca^2+^ regulation in muscle fibers (Lauritzen et al., 2009). The drop-jump exercise is even more demanding because the lengthening of actively contracting muscle fibers is followed immediately by a concentric contraction (Komi, 2000). Because such contractions are high force and high strain, they may cause damage not only to the muscle but also to the tendons and ligaments. It is no surprise that intratendinous air was more frequently found after this type of exercise than after maximum-intensity cycling-induced metabolic fatigue identified by an increase in blood lactate (>12 times) and a large decline in cycling peak power (about 70%). The latter exercise induces metabolic perturbations such as decreased ATP and PCr and accumulation of ADP, AMP, Pi, H^+^, and Mg^2+^ and negatively affects the cross-bridge interaction and calcium shuttling between the sarcoplasmic reticulum and the myoplasm (Ament and Verkerke, 2009), but is much less mechanically demanding in respect of muscle lengthening. We also made the assumption that such a high number of drop jumps might induce signs of pathological changes to the tendons, but we observed only minor pathological changes on ultrasonography, which had disappeared after 24 h.

One of the weaknesses of our study is that the subjects underwent only ultrasound measurements in addition to standard 12 MHz ultrasound device does not allow estimating air bubbles' size accurately. However, it is rather difficult to evaluate such small air accumulations in the tendons using other imaging techniques. We did not estimate air bubbles size consistently but random assessment found that the size of the smallest air bubble was about 190–270 μm. Obviously, during radiographic or CT imaging it is difficult to differentiate such small air bubbles because they overlap with the surrounding tissues. During MRI, the air bubbles are too small to cause susceptibility artifacts, and in addition, the tendon itself appears black, as do the air bubbles (Malghem et al., 2011; Hodgson et al., 2012). Further research is planned to better characterize intratendinous air by obtaining higher quality images.

## Conclusions

The presence of intratendinous air seems related to high-magnitude high-force high-strain exercise at particular tendon areas. It may represent the tendon stress response to overload conditions. Further research is required to establish the possible links between the presence of intratendinous air and the development of tendinopathy, especially for individuals involved in regular eccentric-type exercise.

## Patient consent

Informed consent was obtained from all individual subjects included in the study.

## Ethics statement

This study was carried out in accordance with the recommendations of Kaunas regional biomedical research ethics committee with written informed consent from all subjects. All subjects gave written informed consent in accordance with the Declaration of Helsinki. The protocol was approved by the Kaunas regional biomedical research ethics committee.

## Author contributions

SR, ASk, and SK contributed to the conception, design, analysis and interpretation of the work. VP, DS, MB, ASn, NB, and RR contributed to the acquisition, analysis and interpretation of the data. All authors contributed to the drafting of the manuscript and approved the final version.

### Conflict of interest statement

The authors declare that the research was conducted in the absence of any commercial or financial relationships that could be construed as a potential conflict of interest.
